# Comparative Cardiovascular Outcomes of SGLT2 Inhibitors in Type 2 Diabetes Mellitus: A Network Meta-Analysis of Randomized Controlled Trials

**DOI:** 10.3389/fendo.2022.802992

**Published:** 2022-03-16

**Authors:** Yu Jiang, Pingping Yang, Linghua Fu, Lizhe Sun, Wen Shen, Qinghua Wu

**Affiliations:** ^1^Department of Cardiovascular Medicine, The Second Affiliated Hospital of Nanchang University, Nanchang, China; ^2^Department of Cardiovascular Medicine, The First Affiliated Hospital of Nanchang University, Nanchang, China; ^3^Department of Endocrinology and Metabolism, The Second Affiliated Hospital of Nanchang University, Nanchang, China; ^4^Department of Cardiovascular Medicine, The First Affiliated Hospital of Xi’an Jiaotong University, Xi’an, China

**Keywords:** type 2 diabetes mellitus, SGLT2 inhibitors, cardiovascular events, meta-analysis, empagliflozin

## Abstract

**Background:**

A network meta-analysis of randomized controlled trials (RCTs) was conducted to explore the cardiovascular outcomes of all the kind and dosages of sodium-glucose cotransport-2 (SGLT2) inhibitors in type 2 diabetes mellitus (T2DM) patients.

**Method and Result:**

The Cochrane Library, PubMed, and Embase databases were systematically searched for studies to compare the therapeutic effects of different SGLT2 inhibitors in T2DM patients. The effect measurements estimate chosen were odds ratios (ORs) and their corresponding 95% confidence interval (CI). Forty-seven RCTs involving a total of 70574 participants were eligible for direct and indirect comparisons. In direct comparison, treatment with dapagliflozin 5mg showed significantly lower risk of all-cause mortality compared with treatment with dapagliflozin 2.5mg (OR 0.09, 95% CI 0.01-0.70). According to NMA, interestingly, empagliflozin 10mg/25mg, and canagliflozin 100mg was associated with significantly lower risks of all-cause mortality compared with placebo (OR of 0.70, 95% CI 0.58-0.85; 0.69, 95% CI 0.57-0.84; and 0.83, 95% CI 0.73-0.95, respectively). Compared with placebo, dapagliflozin 10mg, empagliflozin 10mg and 25mg displayed the lower risks for cardiovascular events (OR 0.78, 95% CI 0.44-1.00; OR 0.47, 95% CI 0.22-0.93; and 0.43, 95% CI 0.24-0.74, respectively) by direct comparison. Moreover, canagliflozin 100/300mg showed significantly higher risks of cardiovascular events compared with empagliflozin 10mg (OR of 4.83, 95% CI 1.14-20.46 and 5.31, 95% CI 1.26-22.34, respectively) and empagliflozin 25mg (4.23, 95% CI 1.13-15.83 and 4.65, 95% CI 1.25-17.27, respectively) according to NMA. There were non-significant differences among all interventions in volume depletion in traditional pairwise meta-analysis. While in NMA, canagliflozin 100/300mg were associated with significantly increased risks of volume depletion compared with placebo (OR of 1.47, 95% CI 1.08-1.99 and 2.19, 95% CI 1.66-2.90, respectively).

**Conclusion:**

In the limitations of the NMA, this study showed that empagliflozin might be better than other SGLT2 inhibitors with low risks of all-cause mortality and cardiovascular events in patients with T2DM suggesting the need for *ad hoc* RCTs.

## 1 Introduction

Type 2 diabetes mellitus (T2DM) affects roughly 451 million adults in 2017 worldwide, these figures were expected to increase to almost 700 million by 2045 (1). T2DM is one of the most important risk factors of cardiovascular disease (CVD) (2, 3), the present of both T2DM and CVD is correlated with higher mortality rate despite advances in treatment (4). Enormous studies have shown that glucose lowing therapy failed to reduce the rates of death, although metabolism benefits were shown in these studies (5). In addition, some antihypergycemic agents increase the risk of all-cause mortality and major adverse cardiovascular events (MACEs) in T2DM patients with established CVD or CVD risk factors (6–8). Thus, novel strategies to improve prognosis and reduce mortality in T2DM patients are needed.

Sodium-glucose cotransport-2 (SGLT2) inhibitors, which reduce blood glucose levels in an insulin-independent manner in T2DM patients (9), are correlated with improvement of many metabolic and hemodynamic abnormalities (10). Moreover, it is important to note that SGLT2 inhibition is associated with reduced aortic stiffness (11) and cardiac structure and function improvement (12). Because of the beneficial cardiometabolic/hemodynamic profile induced by SGLT2 inhibitors treatment, clinical studies investigated the efficacy and safety of this class of drugs in T2DM patients (13–15). However, debate continues as to whether all SGLT2 inhibitors that exert similar cardioprotective effects.

Network meta-analysis (NMA) offers the potential to assess multiple therapeutic strategies simultaneously within a single framework and to rank treatments based on efficacy and safety (16). In the current paper, we conducted an NMA of randomized controlled trials (RCTs) for the first time to explore cardiovascular outcomes of different kind and dosages of SGLT2 inhibitors in T2DM patients.

## 2 Methods

### 2.1 Data Sources and Search Strategy

We conducted a systematic search up to October 1, 2020, without any language restriction, using PubMed, Embase, the Cochrane Library, and Clinical trials. We searched studies with key words and Medical Subject Headings that covered “diabetic” or “diabetes” or “Type 2 Diabetes Mellitus” or “T2MD” and “sodium-glucose co-transporter 2 inhibitors”, or “SGLT 2 inhibitors” or “SGLT2 inhibitors” or “sodium-glucose transporter inhibitors” or “canagliflozin” or “dapagliflozin” or “empagliflozin” or “ipragliflozin” or “remogliflozin” or “tofogliflozin” or “sergliflozin”. We also reviewed the corresponding reference list of each retrieved article to identify any relevant studies that may be neglected. The meta-analysis was conducted and reported according to the Preferred Reporting Items of Systematic Reviews and Meta-Analyses (PRISMA) guidelines (17). The search strategies are provided in **Supplementary Table S1**.

### 2.2 Selection Criteria

We collected all RCTs to compare the therapeutic effects of different SGLT2 inhibitors in T2MD patients in this network meta-analysis. Inclusion criteria of the studies were as follows: (a) T2MD patients treatment with SGLT2 inhibitors, (b) study design was an RCT of the treatment group (SGLT2 inhibitors) and control group, (c) studies with outcomes of “all-cause death”, “all-cause mortality”, “myocardial infarction”, “nonfatal myocardial infarction”, “nonfatal stroke”, “cardiovascular death”, “hypertension”, “hypotension”, “volume depletion”, “dehydration”, or “hypovolemia”. The detail was shown in the **Table 1**.

**Table 1:** Inclusion and exclusion criteria.

Category	Inclusion criteria	Exclusion criteria
Patient population	T2DM	T1DM, any other disease and non-human-studiese, GFR < 30 mL/min per 1.73m^2^
Intervention/comparator	SGLT2 inhibitors and control group	Other oral hypoglycemic drugs vs SGLT2 inhibitors
Outcome	“All-cause mortality”, “cardiovascular events” , “volume depletion”	No “all-cause mortality”, “cardiovascular events” , and “volume depletion” outcomes reported
Study design	RCT	Not-RCTs: systemic reviews, comments, case reports, conference abstracts, and editorials
Language	English	Non-English language publications

T2DM, type 2 diabetes mellitus; T1DM, type 1 diabetes mellitus; SGLT2, sodium-glucose cotransport-2; RCT, randomized controlled trials.

The criteria for exclusion were as follows: (a) studies such as systemic reviews, comments, case reports, conference abstracts, and editorials, (b) subjects with an eGFR level lower than 30 mL/min per 1.73m (2), and (c) articles that had no data on T2DM patients.

Included trials reported comparisons of 10 interventions (placebo, dapagliflozin 2.5mg, 5mg, 10mg; empagliflozin 10mg, 25 mg; and canagliflozin 100mg, 300 mg). NMA integrates data from direct comparisons of treatments within trials and from indirect comparisons of interventions assessed against a common comparator in separate trials to compare all investigated treatments.

### 2.3 Data Extraction and Quality Assessment

**Figure 1:**
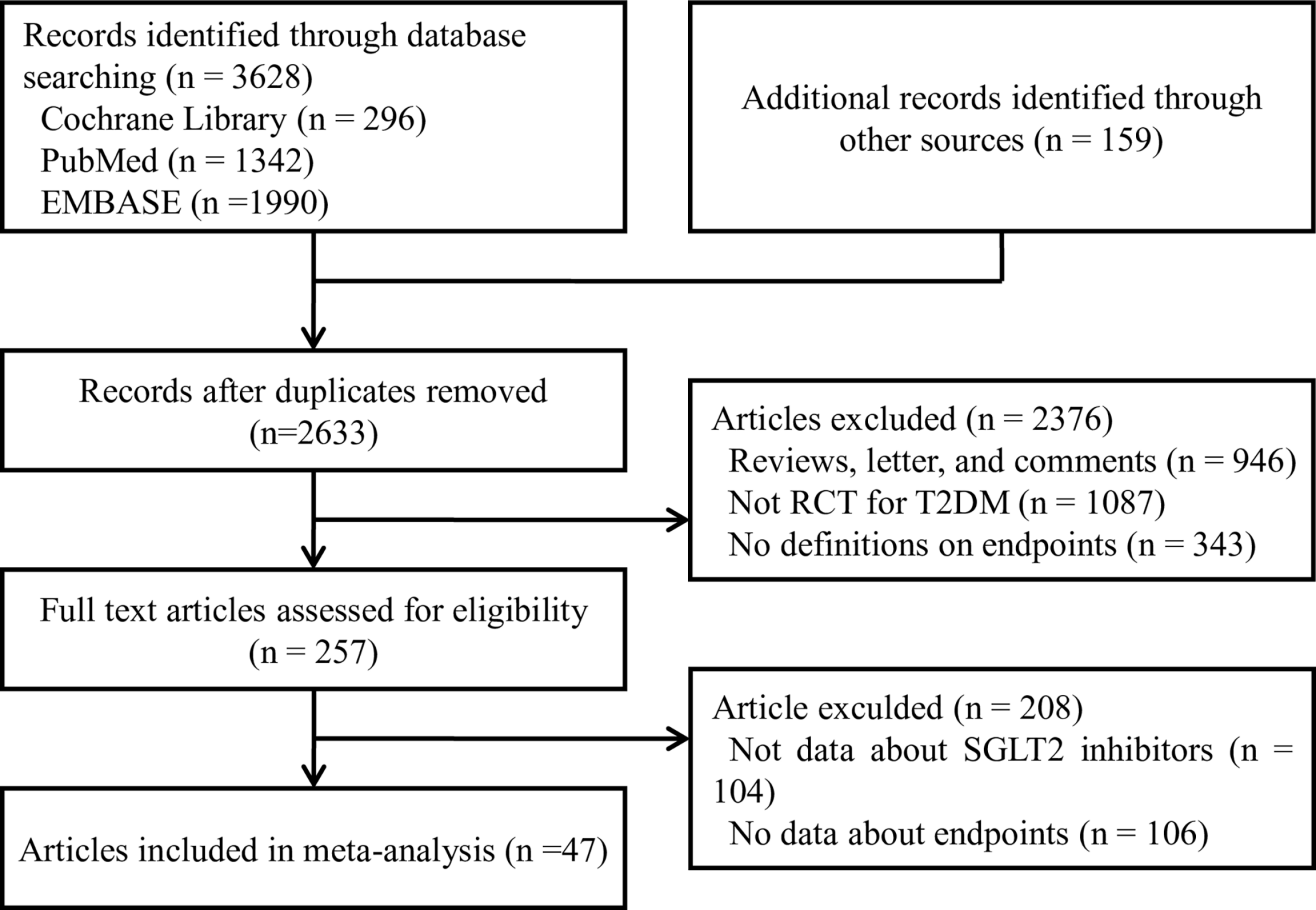
PRISMA diagram of the study selection process for the meta-analysis.

Two authors (J-Y and Y-PP) extracted data and accessed quality independently in an electronic database. The investigators cross-checked the data and reached a consensus on any discrepancies through discussion. Disagreements were resolved through discussions or referral to other authors (S-W and W-QH). Reference lists of identified trials and review articles were manually scanned to identify related research references at the same time as indicated in **Figure 1**.

The extracted data included the first author’s name, year of publication, clinical characteristics, HbA1C% level, sample size, the number of males, doses of treatment, control, combined drugs, follow-up duration, the outcomes of all-cause mortality, cardiovascular events, volume depletion. Cardiovascular events included “myocardial infarction”, “nonfatal myocardial infarction”, “nonfatal stroke”, “cardiovascular death”, and “hypertension”.

### 2.4 Risk of Bias Assessment

Two independent reviewers (J-Y and Y-PP) assessed the methodological quality of included trials using a slightly adapted version of the risk of bias approach by using Review Manager 5.3 (Cochrane Collaboration, Oxford, UK) risk of bias tool including four sections: selection, performance, detection, attrition, reporting, and other bias. The publication bias assessment was performed *via* Deek’s funnel plot asymmetry.

### 2.5 Statistical Analysis

The data were abstracted and analyzed by STATA (version 14.0, Stata MP) and Review Manager (version 5.3, Cochrane Collaboration, Copenhagen, Denmark). The odds ratios (ORs) and their corresponding 95% confidence interval (CI) were used to compare different medications with respect to various clinical outcomes. For each analysis, we generated 50000 simulations for each of the 2 sets of different initial values and discarded the first 20000 simulations as the burn-in period. The stability of the results was obtained by sensitivity analyses by discarding each study sequentially. Convergence was checked using trace plots and the Brooks-Gelman-Rubin (18). To rank the treatments for an outcome, we used the surface under the cumulative ranking area (SUCRA) probabilities (19). Thus, a larger SUCRA score might indicate a higher probability of the end point event. We also used Loop-specific inconsistency (used in Stata and R software) to assess the inconsistency that is the actual difference between direct and indirect comparisons (20).

## 3 Results

### 3.1 Description of Included Studies

We identified 3787 unique records from our searches. Forty-seven RCTs involving a total of 70574 participants were eligible for this NMA. The selection process details are shown in **Figure 1**. The trials included were issued up to September 2020. **Table 2** summarizes the essential baseline characteristics of these included studies **(**detail in **Supplementary Table S2****)**. Of 43 studies, all studies reported the end point event of all-cause death, 25 studies submitted data on cardiovascular events, and 30 studies provided data on volume depletion. The number of patients included in every study ranged from 35 to 17160, and the follow-up for patients ranged from 4 to 201 weeks. The risk of bias in studies contributing to the primary outcomes was generally low (**Supplementary Table S3**).

**Table 2:** Baseline characteristics of included RCTs.

Study	Disease	N	Male	Mean age	HbA1c	SGLT2 inhibitors	Control	Combined drugs	Follow-up	Outcomes
**Bailey (****21****)**	T2DM	546	292	–	8.06%	Dapagliflozin 2.5mg, 5mg, 10mg	Placebo	Metformin	102 weeks	Deaths, CV events, VD
**Bailey (****22****)**	T2DM	274	132	52.2	7.91%	Dapagliflozin 2.5mg, 5mg, 10mg	Placebo	Metformin	102 weeks	Deaths, VD
**Barnett (****23****)**	T2DM+CKD	738	430	63.9	8.00%	Empagliflozin 5mg, 10mg	Placebo	–	52 weeks	Deaths, CV events, VD
**Bolinder (****24****)**	T2DM	180	100	60.7	7.17%	Dapagliflozin 10mg	Placebo	Metformin	24 weeks	Deaths, VD
**Bode (****25****)**	T2DM	714	396	63.6	7.77%	Canagliflozin 100mg, 300mg	Placebo	–	104 weeks	Deaths, CV events, VD
**Davies (****26****)**	T2DM+CVD	2313	1146	55.9	8.00%	Canagliflozin 100mg, 300mg	Placebo	–	26 weeks	Deaths
**DeFronzo (****27****)**	T2DM	674	362	56.2	7.98%	Empagliflozin 10mg, 25mg	Metformin	Linagliptin	52 weeks	Deaths
**Fioretto (****28****)**	T2DM+CKD	321	182	65.8	8.18%	Dapagliflozin 10 mg	Placebo	–	24 weeks	Deaths, VD
**Ferrannini (****29****)**	T2DM	326	172	58.0	7.85%	Empagliflozin 5mg, 10mg, 25mg	Placebo	–	12 weeks	CV events, VD
**Frías (****30****)**	T2DM	685	328	54.3	9.30%	Dapagliflozin 10 mg	Placebo	Exenatide	28 weeks	Deaths, CV events, VD
**Fulcher (****31****)**	T2DM	411	273	62.5	8.09%	Canagliflozin 100mg, 300mg	Placebo	DPP-4i, GLP-1RA	18 weeks	Deaths, VD
**Haring (****32****)**	T2DM	666	390	57.1	8.1%	Empagliflozin 10 mg, 25 mg	Placebo	metformin + sulphonylurea	76 weeks	Deaths, VD
**Henry (****33****)**	T2DM	200	102	56.9	8.34%	Dapagliflozin 10mg	Placebo	insulin + metformin	4 weeks	Deaths
**Inagaki (****34****)**	T2DM	146	93	59.2	8.87%	Canagliflozin 100mg	Placebo	–	18 weeks	Deaths
**Jabbour (****35****)**	T2DM	685	328	54.3	9.31%	Dapagliflozin 10mg	Placebo	Exenatide	52 weeks	Deaths, CV events, VD
**Kadowaki (****36****)**	T2DM	547	410	57.5	7.95%	Empagliflozin 5 mg, 10 mg, 25 mg, 50 mg	Placebo	–	12 weeks	Deaths, VD
**Kaku (****37****)**	T2DM	261	155	58.8	7.50%	Dapagliflozin 5 mg, 10 mg	Placebo	–	24 weeks	CV events,
**Kaku (****38****)**	T2DM with CVD	1517	1118	61.0	8.07%	Empagliflozin 10 mg, 25 mg	Placebo	–	48 weeks	Deaths, CV events
**Kohan (****39****)**	T2DM and MRI	252	164	67.0	8.35%	Dapagliflozin 5mg, 10mg	Placebo	–	104 weeks	Deaths, CV events, VD
**Kovacs (****40****)**	T2DM	498	241	54.5	8.09%	Empagliflozin 10 mg, 25 mg	Placebo	–	76 weeks	Deaths, VD
**Lavalle-González (****41****)**	T2DM	1971	930	55.7	8.1%	Canagliflozin 100 mg, 300 mg	Placebo	Metformin, sulfonylurea	52 weeks	Deaths, VD
**Leiter (****42****)**	T2DM and CVD	1924	1288	63.8	8.05%	Dapagliflozin 10mg	Placebo	–	52 weeks	Deaths, CV events, VD
**Mahaffey (****43****)**	T2DM and CKD	4401	2907	63.0	8.3%	Canagliflozin 100mg	Placebo	–	126 weeks	Deaths, CV events
**Merton (****44****)**	T2DM	2313	1146	55.9	8.0%	Canagliflozin 100 mg, 300 mg	Placebo	–	26 weeks	Deaths, CV events, VD
**Perkovic (****45****)**	T2DM and CKD	4041	2547	63.0	8.3%	Canagliflozin 100 mg	Placebo	–	168 weeks	Deaths, CV events
**Roden (****46****)**	T2DM	899	551	55.0	7.88%	Empagliflozin 10 mg, 25 mg	Placebo	Sitagliptin	76 weeks	Deaths, CV events
**Romera (****47****)**	T2DM and obese	439	247	52.5	8.7%	Empagliflozin 10 mg, 25 mg	Placebo	–	24 weeks	Deaths, VD
**Rosenstock (****48****)**	T2DM	1186	569	54.9	8.8%	Canagliflozin 100mg, 300mg	Placebo	Metformin	26 weeks	Deaths, VD
**Rosenstock (****49****)**	T2DM	424	250	58	7.9%	Empagliflozin 5 mg, 10 mg, 25 mg, 50mg	Placebo	sitagliptin	12 weeks	CV events
**Rosenstock (****50****)**	T2DM	494	276	58.8	8.2%	Empagliflozin 10 mg, 25 mg	Placebo	–	78 weeks	Deaths, CV events
**Schumm-Draeger (****51****)**	T2DM	399	179	57.7	7.8%	Dapagliflozin 2.5mg, 5mg, 10mg	Placebo	Metformin	16 weeks	Deaths, VD
**Sinclair (****52****)**	T2DM	4058	2364	58.2	8.1%	Canagliflozin 100mg, 300mg	Placebo	–	52 weeks	Deaths, VD
**Søfteland (****53****)**	T2DM	327	191	55.2	7.97%	Empagliflozin 10 mg, 25 mg	Placebo	Metformin+ Linagliptin	24 weeks	Deaths
**Stenlo¨f (****54****)**	T2DM	584	258	55.4	8.0%	Canagliflozin 100mg, 300mg	Placebo	–	52 weeks	Deaths, VD
**Strojek (****55****)**	T2DM	592	285	59.8	8.11%	Dapagliflozin 2.5mg, 5mg, 10mg	Placebo	Glimepiride	48 weeks	Deaths, CV events, VD
**Tikkanen (****56****)**	T2DM+ hypertension	823	495	60.2	7.90%	Empagliflozin 10 mg, 25 mg	Placebo	–	12 weeks	Deaths VD
**Tinahones (****57****)**	T2DM	467	254	56.6	7.96%	Empagliflozin 10 mg, 25 mg	Placebo	Metformin+ Linagliptin	24 weeks	Deaths
**Weber 2016 (****58****)**	T2DM+ hypertension	449	247	56.6	8.05%	Dapagliflozin 10mg	Placebo	–	12 weeks	VD
**Wilding (****59****)**	T2DM	469	239	56.8	8.10%	Canagliflozin 100mg, 300mg	Placebo	–	52 weeks	Deaths VD
**Wiviott (****15****)**	T2DM	17160	10738	63.7	8.30%	Dapagliflozin 10mg	Placebo	–	201 weeks	Deaths, CV events, VD
**Yale (****60****)**	T2DM+CKD	269	163	68.5	8.0%	Canagliflozin 100mg, 300mg	Placebo	–	52 weeks	Deaths, VD
**Yale (****61****)**	T2DM	146	79	65.1	8.2%	Canagliflozin 100mg, 300mg	Placebo	–	52 weeks	Deaths, VD
**Yang (****62****)**	T2DM	1453	801	54.7	8.12%	Dapagliflozin 5mg, 10mg	Placebo	–	24 weeks	Deaths, VD
**Yang (****63****)**	T2DM	272	110	57.5	8.56%	Dapagliflozin 10mg	Placebo	Insulin	24 weeks	Deaths, VD
**Zinman (****13****)**	T2DM	7020	5016	63.1	8.7%	Empagliflozin 10mg, 25mg	Placebo	–	136 weeks	Deaths, CV events, VD
**Mordi (****64****)**	T2DM+HF	23	17	69.8	7.9%	Empagliflozin 25mg	Placebo	furosemide	8 weeks	CV events VD
**Eickhoff (****65****)**	T2DM	36	89	64.0	7.5%	Dapagliflozin 10 mg	Placebo	–	24 weeks	Deaths, CV events, VD

RCTs, randomized controlled trial; N, mumble; HbA1c, hemoglobin A1c; SGLT2, Sodium glucose co-transporter 2; T2DM, type 2 diabetes mellitus; CKD, chronic kidney disease; CVD, cardiovascular disease; CV, cardiovascular; VD, volume depletion.

**Figure 2:**
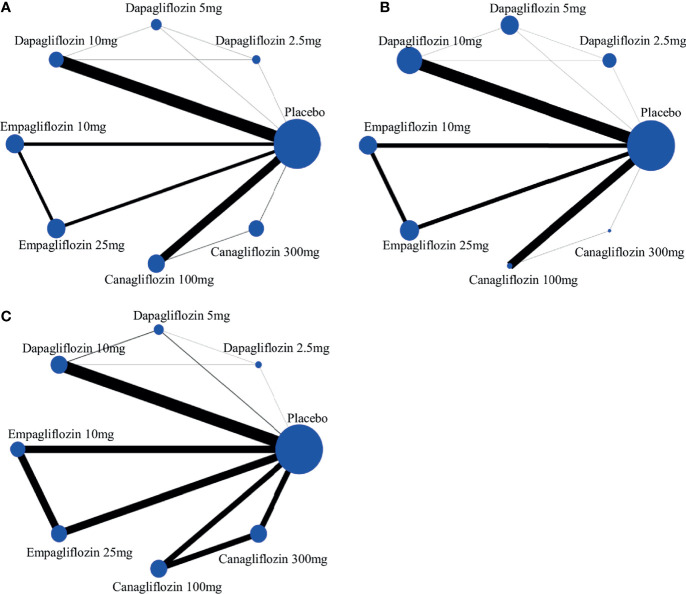
The evidence network of studies reporting **(A)** all-cause death, **(B)** cardiovascular event and **(C)** volume depletion. The size of the nodes (blue circles) corresponds to the overall sample size of the corresponding intervention. Each line represents the direct comparison between the two interventions, and its thickness corresponds to the number of trials that assessed the comparison.

A network plot of treatment comparisons for NMA is shown in **Figure 2**. There are 8 interventions for all-cause death, cardiovascular events and volume depletion. The size of the nodes (blue circles) corresponds to the sample size of the interventions. The comparisons are linked by a straight line, of which the thickness corresponds to the number of trials that assessed the comparison. As shown in the network plot, the number of interventions varied in different subjects.

### 3.2 The Outcome

#### 3.2.1 All-Cause Mortality

We performed a series of traditional pairwise meta-analysis and NMA to summarize the results of trials directly and indirectly comparing the same classes of SGLT2 inhibitors. In direct comparison, there were non-significant differences among all interventions in all-cause mortality, expected Dapagliflozin 5mg vs 2.5mg (OR 0.09, 95% CI 0.01-0.70). According to NMA, interestingly, empagliflozin 10mg/25mg, and canagliflozin 100mg was associated with significantly lower risks of all-cause mortality compared with placebo (OR of 0.70, 95% CI 0.58-0.85; 0.69, 95% CI 0.57-0.84; and 0.83, 95% CI 0.73-0.95, respectively). Moreover, empagliflozin 10mg/25mg was leaded to significantly lower risks of all-cause mortality compared with dapagliflozin 10mg (OR of 0.75, 95% CI 0.60-0.95; 0.74, 95% CI 0.59-0.93, respectively), as depicted in **Table 3A**.

**Table 3A:** Summary of results from network meta-analysis and traditional pairwise meta-analysis on all-cause death.

OR 95% CI	Placebo	Dapagliflozin 2.5mg	Dapagliflozin 5mg	Dapagliflozin 10mg	Empagliflozin 10mg	Empagliflozin 25mg	Canagliflozin 100mg	Canagliflozin 300mg
**Placebo**		3.10 (0.26, 47.00)	0.27 (0.04, 2.70)	0.94 (0.55, 1.70)	0.60 (0.22, 1.70)	0.70 (0.32, 1.70)	0.82 (0.53, 1.20)	0.56 (0.12, 2.20)
**Dapagliflozin 2.5mg**	1.67 (0.56, 5.01)		**0.09 (0.01, 0.70)**	0.41 (0.01, 4.30)				
**Dapagliflozin 5mg**	0.66 (0.24, 1.81)	0.40 (0.10, 1.50)		1.50 (0.13, 17.00)				
**Dapagliflozin 10mg**	0.93 (0.83, 1.04)	0.56 (0.19,1.67)	1.40 (0.51, 3.85)					
**Empagliflozin 10mg**	**0.70 (0.58, 0.85)**	0.42 (0.14, 1.28)	1.06 (0.38, 2.96)	**0.75 (0.60, 0.95)**				
**Empagliflozin 25mg**	**0.69 (0.57, 0.84)**	0.41 (0.14, 1.26)	1.04 (0.37, 2.91)	**0.74 (0.59, 0.93)**	0.98 (0.79, 1.22)			
**Canagliflozin 100mg**	**0.83 (0.73, 0.95)**	0.50 (0.16,1.50)	1.25 (0.45, 3.46)	0.89 (0.75, 1.06)	1.18 (0.93, 1.50)	1.20 (0.95, 1.53)		
**Canagliflozin 300mg**	0.66 (0.40,1.08)	0.39 (0.12,1.31)	0.99 (0.32, 3.05)	0.71 (0.43, 1.18)	0.94 (0.55, 1.60)	0.95 (0.56, 1.63)	0.79 (0.48, 1.31)	

The comparative effects of different class and doses of SGLT2 inhibitors in reducing mortality by SUCRA probabilities and incidence rate of each intervention was shown in **Table 4**. The NMA suggested that higher dosage of empagliflozin (25 mg once daily) was associated with the lowest probability of achieving at all-cause mortality (SUCRA, 23.9%), followed by canagliflozin 300 mg (SUCRA, 24.4%), and empagliflozin 10 mg (SUCRA, 26.3%). However, dapagliflozin 2.5mg was associated with the highest probability of all-cause death (SUCRA, 89.7%).

#### 3.2.2 Cardiovascular Events

In traditional pairwise meta-analysis, compared with placebo, dapagliflozin 10mg, empagliflozin 10mg and 25mg displayed the lowest risks for cardiovascular events (OR 0.78, 95% CI 0.44-1.00; OR 0.47, 95% CI 0.22-0.93; and 0.43, 95% CI 0.24-0.74, respectively). According to NMA, canagliflozin 100/300mg was associated with significantly higher risks of cardiovascular events compared with empagliflozin 10mg (OR of 4.83, 95% CI 1.14-20.46 and 5.31, 95% CI 1.26-22.34, respectively) and empagliflozin 25mg (4.23, 95% CI 1.13-15.83 and 4.65, 95% CI 1.25-17.27, respectively) (**Table 3B**). Empagliflozin 25mg and dapagliflozin 5mg ranked the best and second (SUCRA of 21.8% and 23.9%, respectively), followed by empagliflozin 10mg (SUCRA of 30.9%). In addition, canagliflozin 300 mg was ranked the least effective treatment in reducing cardiovascular events (**Table 4**).

**Table 3B:** Summary of results from network meta-analysis and traditional pairwise meta-analysis on cardiovascular events.

OR 95% CI	Placebo	Dapagliflozin 2.5mg	Dapagliflozin 5mg	Dapagliflozin 10mg	Empagliflozin 10mg	Empagliflozin 25mg	Canagliflozin 100mg	Canagliflozin 300mg
**Placebo**		0.67 (0.23, 2.00)	0.51 (0.19, 1.40)	0.78 (0.44, 1.00)	0.47 (0.22,0.93)	0.43 (0.24, 0.74)	0.75 (0.53, 1.40)	4.0 (0.77,21.00)
**Dapagliflozin 2.5mg**	0.30 (0.03, 2.57)		0.55 (0.10, 3.10)	0.64 (0.12, 3.30)				
**Dapagliflozin 5mg**	1.19 (0.30, 4.76)	4.04 (0.42, 39.15)		0.87 (0.22, 3.50)				
**Dapagliflozin 10mg**	1.14 (0.42, 3.09)	3.86 (0.44, 33.53)	0.96 (0.24, 3.81)					
**Empagliflozin 10mg**	0.66 (0.31, 1.40)	2.24 (0.23, 22.01)	0.55 (0.12, 2.61)	0.58 (0.17, 2.01)				
**Empagliflozin 25mg**	0.76 (0.47, 1.21)	2.56 (0.28, 23.37)	0.63 (0.15, 2.71)	0.66 (0.22, 2.00)	1.14 (0.60, 2.19)			
**Canagliflozin 100mg**	3.20 (0.93, 10.98)	10.81 (0.90, 130.36)	2.68 (0.42, 17.10)	2.80 (0.57, 13.70)	**4.83 (1.14, 20.46)**	**4.23 (1.13, 15.83)**		
**Canagliflozin 300mg**	3.51 (1.03, 11.97)	11.89 (0.99, 142.75)	2.94 (0.46, 18.70)	3.08 (0.63, 14.97)	**5.31 (1.26, 22.34)**	4.65 (1.25, 17.27)	1.10 (0.44, 2.75)	

#### 3.2.3 Volume Depletion

There were non-significant differences among all interventions in volume depletion in traditional pairwise meta-analysis. While in NMA, canagliflozin 100/300mg were associated with significantly increased risks of volume depletion compared with placebo (OR of 1.47, 95% CI 1.08-1.99 and 2.19, 95% CI 1.66-2.90, respectively). The incidence of volume depletion induced by canagliflozin 300mg was significantly higher than that induced by dapagliflozin 5/10mg, empagliflozin 10/25mg and canagliflozin 100mg. These NMA results are illustrated in **Table 3C**.

**Table 3C:** Summary of results from network meta-analysis and traditional pairwise meta-analysis on volume depletion.

OR 95% CI	Placebo	Dapagliflozin 2.5mg	Dapagliflozin 5mg	Dapagliflozin 10mg	Empagliflozin 10mg	Empagliflozin 25mg	Canagliflozin 100mg	Canagliflozin 300mg
**Placebo**		0.027 (0.01, 67.00	1.60 (0.39, 6.80)	1.20 (0.84, 2.30)	1.10 (0.45, 2.60)	1.30 (0.62, 2.70)	1.70 (0.78, 3.90)	2.50 (1.30, 4.90)
**Dapagliflozin 2.5mg**	1.36 (0.24, 7.55)		0.01 (0.00, 14.00	1.20 (0.03, 42.00)				
**Dapagliflozin 5mg**	0.94 (0.44,2.00)	0.69 (0.12, 4.18)		1.20 (0.37, 3.70)				
**Dapagliflozin 10mg**	1.06 (0.89,1.27)	0.78 (0.14, 4.34)	1.12 (0.53, 2.36)					
**Empagliflozin 10mg**	1.02 (0.81, 1.28)	0.75 (0.13, 4.26)	1.08 (0.49, 2.38)	0.96 (0.72, 1.29)		1.10 (0.48, 2.40)		
**Empagliflozin 25mg**	1.13 (0.90, 1.41)	0.83 (0.15, 4.71)	1.20 (0.55, 2.62)	1.07 (0.80, 1.42)	1.10 (0.89, 1.38)			
**Canagliflozin 100mg**	**1.47 (1.08, 1.99)**	1.08 (0.19, 6.19)	1.56 (0.69, 3.50)	1.39 (0.97, 1.97)	1.44 (0.98, 2.10)	1.30 (0.89, 1.89)		
**Canagliflozin 300mg**	**2.19 (1.66, 2.90)**	1.62 (0.28, 9.22)	**2.33 (1.04, 5.19)**	**2.07 (1.49, 2.89)**	**2.15 (1.50, 3.08)**	**1.94 (1.36, 2.78)**	**1.50 (1.16, 1.92)**	

Summary of results from network meta-analysis and traditional pairwise meta-analysis on all-cause death.On the lower triangle, the column-defining treatment is compared to the row-defining treatment, and odds ratios (OR) < 1 favor the column-defining treatment. On the upper triangle, the row-defining treatment is compared to the column-defining treatment, and OR < 1 favor the row-defining treatment. To obtain ORs for comparisons in the opposite direction, reciprocals should be taken. To obtain ORs for comparisons in the opposite direction, reciprocals should be taken. Significant results are in bold.

Canagliflozin 300mg had the highest probabilities of being ranked first with respect to volume depletion (SUCRA 95.5%), whereas canagliflozin 100mg had the second highest probability (SUCRA 75.1%). Both dapagliflozin 5mg and empagliflozin 10mg shown smallest cumulative probabilities for volume depletion, with all values lower 30% (SUCRA 29.9% and 30.5%, respectively), as depicted in **Table 4**.

**Table 4:** Incidence rate and SUCRA for the efficacy of treatments to induce end points in the T2DM patients.

End event	All-cause death				Cardiovascular events				Volume depletion			
Treatment	Event (n)	Total (n)	Incidence rate (%)	SUCRA (%)	Event (n)	Total (n)	Incidence rate (%)	SUCRA (%)	Event (n)	Total (n)	Incidence rate (%)	SUCRA (%)
**Placebo**	1027	20916	4.91	83.0	1814	16162	11.22	80.0	284	15524	1.83	25.0
**Dapagliflozin 2.5mg**	4	456	0.87	89.7	12	456	2.63	46.9	1	456	0.22	55.0
**Dapagliflozin 5mg**	3	1020	0.29	33.7	13	615	2.11	23.9	11	1020	1.08	29.9
**Dapagliflozin 10mg**	539	10484	5.14	68.4	27	1822	1.48	45.7	245	11207	2.19	38.6
**Empagliflozin 10mg**	17	2534	0.67	26.3	24	902	2.66	30.9	29	1287	2.25	30.5
**Empagliflozin 25mg**	21	2692	0.78	23.9	35	1108	3.16	21.8	41	1488	2.76	50.5
**Canagliflozin 100mg**	347	8128	4.27	50.6	559	4645	12.03	63.1	41	2814	1.46	75.1
**Canagliflozin 300mg**	5	3648	0.14	24.4	14	236	5.93	87.7	62	2814	2.20	95.5

The graphs display the distribution of probabilities of treatment ranked from best to worst for each outcome. The ranking indicates the probability that the drug class is first “best,” second “best”, etc. For example, the ranking suggests that dapagliflozin 2.5mg posed the highest risk for incurring all-cause death (worst), while empagliflozin 25mg incurred the lowest probability of all-cause death (best).

### 3.3 Exploration of Inconsistency, Sensitivity Analysis, and Publication Bias

Treatment from network meta-analysis evidence in general did not demonstrate evidence of statistical inconsistency (**Supplementary Figure S1**). As shown in **Supplementary Figure S1**, the inconsistency plot consists of two triangular loops and four quadrangular loops. The IF values of all loops were truncated at zero, and P value > 0.05 verified their consistency statistically.

A sensitivity analysis was conducted to examine the impact of studies according to the treatment effects on the outcomes of all-cause death, cardiovascular events, and volume depletion. We performed an analysis in T2DM used Bayesian, and there was no significant difference in the different methods (**Supplementary Table S3**). No significant publication bias was detected in the funnel plot (**Supplementary Figure S2**).

## 4 Discussion

In recent years, clinical trials revealed the cardioprotective effects of SGLT2 inhibitors in T2DM patients (13–15). Moreover, SGLT-2 inhibitors were most likely to rank best for all-cause/cardiac mortality and the outcomes of heart failure and myocardial infarction compared with dipeptidyl peptidase 4 inhibitors and glucagon-like peptide 1 agonists (66). However, no studies have directly or simultaneously compared the efficacy and safety of all the kind and dosages of SGLT2 inhibitors. In the present meta-analysis, we grouped all available SGLT2 inhibitors together, including dapagliflozin 2.5mg/5mg/10mg, empagliflozin 10mg/25mg, and canagliflozin 100mg/300mg. Since only few studies allowed direct comparisons, indirect comparisons were further conducted to identified the effects of different SGLT2 inhibitors on all-cause mortality, cardiovascular events and volume depletion, respectively. We demonstrated that dose of dapagliflozin 5mg was associated with all-cause mortality reduction, rather than other SGLT2 inhibitors by direct comparison. According to NMA, empagliflozin 10mg/25mg, and canagliflozin 100mg was associated with significantly lower risks of all-cause mortality compared with placebo. Moreover, empagliflozin 10mg/25mg was leaded to significantly lower risks of all-cause mortality compared with dapagliflozin 10mg. Dapagliflozin 10mg, empagliflozin 10mg and 25mg displayed the lower risks for cardiovascular events compared with placebo. In addition, it seems likely that canagliflozin 100/300mg showed significantly higher risks of cardiovascular events compared with empagliflozin 10mg/25mg according to NMA. Finally, we suggested that treatment with canagliflozin 100/300mg were associated with significantly increased risks of volume depletion compared with placebo by NMA.

In recent years, numerous studies investigated the role of SGLT2 inhibitor on mortality reduction. For instance, In EMPA-REG OUTCOME trial, Zinman and coworkers revealed that the rate of all-cause mortality was significantly lower in patients who received empagliflozin 10mg/25mg than controlled group (13). More recently, however, CANVAS (Canagliflozin Cardiovascular Assessment Study) Program which is an integrated analysis of CANVAS and CANVAS-R (Canagliflozin Cardiovascular Assessment Study-Renal) (14), and DECLARE–TIMI 58 trial (15) failed to show the positive effect of canagliflozin 100mg/300 mg and dapagliflozin 10mg on reducing the rate of all-cause death, respectively. The major limitations of CANVAS Program are lack of events number and discontinuation of randomized therapy, which may result in underestimation of benefits effect of canagliflozin on all-cause mortality reduction. The present meta-analysis grouped all available SGLT2 inhibitors for direct and indirect comparison. Since only few studies allowed direct comparisons between SGLT2 inhibitors, NMA were conducted and found that dapagliflozin 5mg was associated with lower risk of all-cause mortality. Moreover, the positive role of empagliflozin 10mg/25mg and canagliflozin 100mg on reducing all-cause mortality was demonstrated by NMA (as shown in **Table 3**). Although the potential factors that responsible for mortality reduction of canagliflozin are need to be further investigated, it may be relevant to identify that canagliflozin has less SGLT2 selectively and may induce more glucosuria (67), suggesting great volume depletion by canagliflozin and rise in hematocrit and hemoconcentration could increase blood viscosity. On the other hand, it has been reported that canagliflozin impacts activation of AMP-kinase in mitochondrial function, which is helpful to keep the balance of cellular energy metabolism (68). As an alternative explanation, we selected 43 studies investigating the efficacy of SGLT2 inhibitors on mortality rate in 66819 T2DM patients and included a total of 2470 deaths, data reporting may have differed between these studies. These finding highlighted that more clinical trials are urgently needed to explore the specific cardioprotective role of SGLT2 inhibitors in T2DM patients, even though they have similar effects.

For patients with T2DM, an important goal of currently treatment is reducing the rate of cardiovascular events. In recent years, clinical trials investigated the role of SGLT2 inhibitors on MACE and suggested that the particular clinical benefits of SGLT2 inhibition may rely on the baseline characteristics of patient population. For instance, the positive effect of empagliflozin on cardiovascular mortality and MACEs rate was revealed in EMPA-REG OUTCOME trial which included T2DM patients with established CVD (13). However, dapagliflozin treatment resulted in lower rate of cardiovascular death rather than total MACEs in DECLARE-TIMI 58 trial which included 41.64% T2DM with CVD patients (15). Moreover, canagliflozin significantly reduce the rate of MACEs in CANVAS Program which included 66% T2DM with CVD patients (14). Recently, a meta-analysis included these three clinical trials and their secondary analyses revealed that SGLT2 inhibitors reduce MACEs by 11% in patients with established CVD. Moreover, they found that the effect of empagliflozin on cardiovascular death was more pronounced than that of canagliflozin or dapagliflozin (69). In currently paper, we suggested that dapagliflozin 10mg, empagliflozin 10mg/25mg and canagliflozin 100mg could exert similar role in reducing the risk of cardiovascular events in T2DM patients, highlighting that SGLT2 inhibitors may be considered to manage T2DM in patients not only with established CVD but also in patients without CVD but at elevated risk. Additionally, we found that empagliflozin 10mg/25mg rank the best and second choice in regarding to cardiovascular events risk reduction by SUCRA probabilities.

It has been reported that SGLT2 inhibition decrease sodium reabsorption and increases urinary sodium excretion (70). In addition to exert multiple metabolic effects including reduce HbA1c, change caloric balance and weight loss, the glucosuria/natriuretic effects of SGLT2 inhibitors are also account for, at least in part, the positive role of it on reducing the rate of hospitalization for heart failure. However, it is important to recognize that these effects may have protective and injurious potential. For instance, natriuresis may also result in increasing risk of volume depletion, such as hypotension and syncope, and promoting neurohormonal activation and tissue ischemia in the periphery (71). According to our analysis, we found that higher dosage of canagliflozin (300 mg once daily) was associated with increased the rate of volume depletion among all SGLT2 inhibitors included in this study, suggesting that the potential of higher dosage of canagliflozin induced volume depletion may account for the neutralize results in all-cause mortality.

We acknowledge several limitations of our study. First, we used aggregated study-level data rather than individual participant data, and the different studies have been conducted in different population. Second, we could not include data from several trials such as DECLARE-TIMI 58, CANVAS Program, EMPA-REG OUTCOME due to data was limited in these studies. Third, we did not perform a subgroup analysis of cardiovascular mortality caused by SGLT2 inhibitors in T2DM patients. Then, although the heterogeneity in the network analysis was low, it is likely that the low power to detect heterogeneity is due to limited data for some dosage SGLT2 inhibitors. Finally, the NMA results do not meet the assumptions of homogeneity of direct evidence and/or transitivity, therefore, several larger multi-center RCTs which investigated effects between SGLT2 inhibitors including patients with similar clinical characteristics need to be implemented to achieve more robust results.

## 5 Conclusion

In the limitations of the NMA, this study showed that empagliflozin 10mg/25mg once daily might be better than other SGLT2 inhibitors with low risks of all-cause mortality and cardiovascular events in patients with T2DM suggesting the need for *ad hoc* RCTs.

## Data Availability Statement

The original contributions presented in the study are included in the article/**Supplementary Material**. Further inquiries can be directed to the corresponding authors.

## Author Contributions

YJ, PY, and LF performed the meta-analysis. LS was responsible for the statistical analysis. WS provided editing assistance. QW prepared the manuscript. All authors contributed to the article and approved the submitted version.

## Funding

This work was supported by the National Natural Science Foundation of China (No. 82160093), the incubation project of the National Natural Science Foundation of the Second Affiliated Hospital of Nanchang University (No. 2021YNFY2021), the Jiangxi Graduate Innovation Special Fund (No. YC2019-B039), the Jiangxi Provincial Health Commission Project (No. 202210663), and the State Scholarship Fund of the China Scholarship Council (grant number 201906820003).

## Conflict of Interest

The authors declare that the research was conducted in the absence of any commercial or financial relationships that could be construed as a potential conflict of interest.

## Publisher’s Note

All claims expressed in this article are solely those of the authors and do not necessarily represent those of their affiliated organizations, or those of the publisher, the editors and the reviewers. Any product that may be evaluated in this article, or claim that may be made by its manufacturer, is not guaranteed or endorsed by the publisher.

## Abbreviations

RCTs, Randomized controlled trials; SGLT2, Sodium-glucose cotransport-2; T2DM, Type 2 diabetes mellitus; ORs, Odds ratios; CVD, Cardiovascular disease; MACEs, Major adverse cardiovascular events; NMA, Network meta-analysis; SUCRA, The surface under the cumulative ranking area; CI, Confidence interval; CANVAS, Canagliflozin cardiovascular assessment study.
