# Supplementary material for: Comparative Cardiovascular Outcomes of SGLT2 Inhibitors in Type 2 Diabetes Mellitus: A Network Meta-Analysis of Randomized Controlled Trials
Source: Front Endocrinol (Lausanne). 2022 Mar 16;13:802992. doi: 10.3389/fendo.2022.802992 (PMC8967154; doi:10.3389/fendo.2022.802992)
Supplement: Supplementary file 1 [file DataSheet_1.doc]

**Title Page**

**Comparative Cardiovascular Outcomes of SGLT2 inhibitors in Type 2 Diabetes Mellites: A Network Meta-analysis of Randomized Controlled Trials**

Yu Jiang,^1#^ Pingping Yang,^1#^ Linghua Fu,^1^ Lizhe Sun,^2^ Wen Shen^1^*, Qinghua Wu^1^*

^1^ Department of Cardiovascular Medicine, the Second Affiliated Hospital of Nanchang University, Nanchang, Jiangxi, China

^2^ Department of Cardiovascular Medicine, the First Affiliated Hospital of Xi'an Jiaotong University. Xi'an, China

*Corresponding Author**:** Wen Shen (Ph.D. & M.D.) and Qinghua Wu (Ph.D. & M.D.)

Department of Cardiovascular Medicine, the Second Affiliated Hospital of Nanchang University, Nanchang, Jiangxi, China; Zip Code: 330006; Address: No. 1, Minde Road, Donghu District, Nanchang, P.R. China; E-mail: 18146622197@163.com and ncwqh@163.com; Tel: +86(0)791 86263601, Fax: +86(0)791 86263601.

# Yu Jiang and Pingping Yang contributed equally to this work

**Short Title:** NMA of SGLT2 to improve T2DM

**Supplementary**

**List of Supplementary**

**Supplementary Table S1:** Electronic search strategies determined on 1^st^ October 2020

**Supplementary Table S2:** Baseline characteristics of included RCTs

**Supplementary Table S3:** Risk of bias of included studies

**Supplementary** **Figure S1**: Inconsistency

**Figure S1A:** Inconsistency for all-cause death

**Figure S1B:** Inconsistency for cardiovascular events

**Figure S1C:** Inconsistency for volume depletion

**Supplementary** **Figure S2**: Publication bias of funnel plot

**Figure S2A:** funnel plot for all-cause death

**Figure S2B:** funnel plot for cardiovascular events

**Figure S2C:** funnel plot for volume depletion

| **Search** | **Query** |
| --- | --- |
| **The Cochrane library** |  |
| #1 | (diabetes) OR (diabetic) OR (diabetes mellitus) OR (type 2 diabetes mellitus) OR (T2MD) in All Text |
| #2 | (canagliflozin) OR (dapagliflozin) OR (empagliflozin) OR (ipragliflozin) OR (remogliflozin) OR (tofogliflozin) OR (sergliflozin) OR (SGLT 2 inhibitors) OR (sodium-glucose co-transporter 2 inhibitors) OR (sodium glucose co-transporter-2 inhibitors) OR (sodium-glucose transporter inhibitors) OR (SGLT2 inhibitors) in All Text |
| #3 | (randomized) OR (randomized controlled trial) OR (controlled) OR (RCT) OR (RCTs) in All Text |
| #4 | #1 and #2 and #3 |
| **PubMed** |  |
| #1 | diabetes OR diabetic OR diabetes mellitus OR type 2 diabetes mellitus OR T2MD |
| #2 | canagliflozin OR dapagliflozin OR empagliflozin OR ipragliflozin OR remogliflozin OR tofogliflozin OR sergliflozin OR SGLT 2 inhibitors OR sodium-glucose co-transporter 2 inhibitors OR sodium glucose co-transporter-2 inhibitors OR sodium-glucose transporter inhibitors OR SGLT2 inhibitors |
| #3 | randomized OR randomized controlled trial OR controlled OR RCT OR RCTs |
| #4 | #1 and #2 and #3 |
| **EMBASE** |  |
| #1 | (diabetes) OR (diabetic) OR (diabetes mellitus) OR (type 2 diabetes mellitus) OR (T2MD) |
| #2 | (canagliflozin) OR (dapagliflozin) OR (empagliflozin) OR (ipragliflozin) OR (remogliflozin) OR (tofogliflozin) OR (sergliflozin) OR (SGLT 2 inhibitors) OR (sodium-glucose co-transporter 2 inhibitors) OR (sodium glucose co-transporter-2 inhibitors) OR (sodium-glucose transporter inhibitors) OR (SGLT2 inhibitors) |
| #3 | (randomized) OR (randomized controlled trial) OR (controlled) OR (RCT) OR (RCTs) |
| #4 | #1 and #2and #3 |

Table S1. Electronic search strategies determined on 1st October 2020

Table S2. Baseline characteristics of included RCTs

| **Study** | **Disease** | **N** | **Male** | **Mean age** | **HbA1C%** | | **SGLT2 inhibitors** | **Control** | **Combined drugs** | **Follow-up** | **Outcomes** |
| --- | --- | --- | --- | --- | --- | --- | --- | --- | --- | --- | --- |
| **Bailey 2013**[**^1^**](#_ENREF_1) | T2DM | 546 | 292 | - | 8.06% | Dapagliflozin 2.5mg, 5mg, 10mg | | Placebo | Metformin | 102 weeks | Deaths, cardiovascular events, volume depletion |
| **Bailey 2015**[**^2^**](#_ENREF_2) | T2DM | 274 | 132 | 52.2 | 7.91% | Dapagliflozin 2.5mg, 5mg, 10mg | | Placebo | Metformin | 102 weeks | Deaths, volume depletion |
| **Barnett 2014**[**^3^**](#_ENREF_3) | T2DM with CKD | 738 | 430 | 63.9 | 8.00% | Empagliflozin 5mg, 10mg | | Placebo | - | 52 weeks | Deaths, cardiovascular events, volume depletion |
| **Bolinder 2012**[**^4^**](#_ENREF_4) | T2DM | 180 | 100 | 60.7 | 7.17% | Dapagliflozin 10mg | | Placebo | Metformin | 24 weeks | Deaths, volume depletion |
| **Bode 2015**[**^5^**](#_ENREF_5) | T2DM | 714 | 396 | 63.6 | 7.77% | Canagliflozin 100mg, 300mg | | Placebo | - | 104 weeks | Deaths, cardiovascular events, volume depletion |
| **Davies 2017**[**^6^**](#_ENREF_6) | T2DM with CVD | 2313 | 1146 | 55.9 | 8.00% | Canagliflozin 100mg, 300mg | | Placebo | - | 26 weeks | Deaths |
| **DeFronzo 2015**[**^7^**](#_ENREF_7) | T2DM | 674 | 362 | 56.2 | 7.98% | Empagliflozin 10mg, 25mg | | Metformin | Linagliptin | 52 weeks | Deaths |
| **Fioretto 2018**[**^8^**](#_ENREF_8) | T2DM with CKD3A | 321 | 182 | 65.8 | 8.18% | Dapagliflozin 10 mg | | Placebo | - | 24 weeks | Deaths, volume depletion |
| **Ferrannini 2013**[**^9^**](#_ENREF_9) | T2DM | 326 | 172 | 58.0 | 7.85% | Empagliflozin 5mg, 10mg, 25mg | | Placebo | - | 12 weeks | Cardiovascular events, Volume depletion |
| **Frías 2016**[**^10^**](#_ENREF_10) | T2DM | 685 | 328 | 54.3 | 9.30% | Dapagliflozin 10 mg | | Placebo | Exenatide | 28 weeks | Deaths, cardiovascular events, volume depletion |
| **Fulcher 2016**[**^11^**](#_ENREF_11) | T2DM | 411 | 273 | 62.5 | 8.09% | Canagliﬂozin 100mg, 300mg | | Placebo | DPP-4 inhibitor,  GLP-1 receptor agonist | 18 weeks | Deaths, volume depletion |
| **Haring 2015**[**^12^**](#_ENREF_12) | T2DM | 666 | 390 | 57.1 | 8.1% | Empagliflozin 10 mg, 25 mg | | Placebo | metformin + sulphonylurea | 76 weeks | Deaths, volume depletion |
| **Henry 2018**[**^13^**](#_ENREF_13) | T2DM | 200 | 102 | 56.9 | 8.34% | Dapagliflozin 10mg | | Placebo | insulin + metformin | 4 weeks | Deaths |
| **Inagaki 2016**[**^14^**](#_ENREF_14) | T2DM | 146 | 93 | 59.2 | 8.87% | Canagliﬂozin 100mg | | Placebo | - | 18 weeks | Deaths |
| **Jabbour 2018**[**^15^**](#_ENREF_15) | T2DM | 685 | 328 | 54.3 | 9.31% | Dapagliflozin 10mg | | Placebo | Exenatide | 52 weeks | Deaths, cardiovascular events, volume depletion |
| **Kadowaki 2014**[**^16^**](#_ENREF_16) | T2DM | 547 | 410 | 57.5 | 7.95% | Empagliflozin 5 mg, 10 mg, 25 mg, 50 mg | | Placebo | - | 12 weeks | Deaths, volume depletion |
| **Kaku 2014**[**^17^**](#_ENREF_17) | T2DM | 261 | 155 | 58.8 | 7.50% | Dapagliflozin 5 mg, 10 mg | | Placebo | - | 24 weeks | Cardiovascular events, |
| **Kaku 2017**[**^18^**](#_ENREF_18) | T2DM with CVD | 1517 | 1118 | 61.0 | 8.07% | Empagliflozin 10 mg, 25 mg | | Placebo | - | 48 weeks | Deaths, cardiovascular events |
| **Kohan 2014**[**^19^**](#_ENREF_19) | T2DM and MRI | 252 | 164 | 67.0 | 8.35% | Dapagliflozin 5mg, 10mg | | Placebo | - | 104 weeks | Deaths, cardiovascular events, volume depletion |
| **Kovacs 2015**[**^20^**](#_ENREF_20) | T2DM | 498 | 241 | 54.5 | 8.09% | Empagliflozin 10 mg, 25 mg | | Placebo | - | 76 weeks | Deaths, volume depletion |
| **Lavalle-González 2016**[**^21^**](#_ENREF_21) | T2DM | 1971 | 930 | 55.7 | 8.1% | Canagliﬂozin 100 mg, 300 mg | | Placebo | Metformin, sulfonylurea | 52 weeks | Deaths, volume depletion |
| **Leiter 2014**[**^22^**](#_ENREF_22) | T2DM and CVD | 1924 | 1288 | 63.8 | 8.05% | Dapagliflozin 10mg | | Placebo | - | 52 weeks | Deaths, cardiovascular events, volume depletion |
| **Mahaffey 2019**[**^23^**](#_ENREF_23) | T2DM and CKD | 4401 | 2907 | 63.0 | 8.3% | Canagliﬂozin 100mg | | Placebo | - | 126 weeks | Deaths, cardiovascular events |
| **Merton 2018**[**^24^**](#_ENREF_24) | T2DM | 2313 | 1146 | 55.9 | 8.0% | Canagliﬂozin 100mg, 300mg | | Placebo | - | 26 weeks | Deaths, cardiovascular events, volume depletion |
| **Perkovic 2019**[**^25^**](#_ENREF_25) | T2DM and CKD | 4041 | 2547 | 63.0 | 8.3% | Canagliﬂozin 100mg | | Placebo | - | 168 weeks | Deaths, cardiovascular events |
| **Roden 2015**[**^26^**](#_ENREF_26) | T2DM | 899 | 551 | 55.0 | 7.88% | Empagliflozin 10 mg, 25 mg | | Placebo | Sitagliptin | 76 weeks | Deaths, cardiovascular events |
| **Romera 2016**[**^27^**](#_ENREF_27) | T2DM and obese | 439 | 247 | 52.5 | 8.7% | Empagliflozin 10 mg, 25 mg | | Placebo | - | 24 weeks | Deaths, volume depletion |
| **Rosenstock 2016**[**^28^**](#_ENREF_28) | T2DM | 1186 | 569 | 54.9 | 8.8% | Canagliﬂozin 100mg, 300mg | | Placebo | Metformin | 26 weeks | Deaths, volume depletion |
| **Rosenstock 2013**[**^29^**](#_ENREF_29) | T2DM | 424 | 250 | 58 | 7.9% | Empagliflozin 1 mg, 5 mg, 10 mg, 25 mg, 50mg | | Placebo sitagliptin | - | 12 weeks | Cardiovascular events |
| **Rosenstock 2015**[**^30^**](#_ENREF_30) | T2DM | 494 | 276 | 58.8 | 8.2% | Empagliflozin 10 mg, 25 mg | | Placebo | - | 78 weeks | Deaths, cardiovascular events |
| **Schumm-Draeger 2016**[**^31^**](#_ENREF_31) | T2DM | 399 | 179 | 57.7 | 7.8% | Dapagliflozin 2.5mg, 5mg, 10mg | | Placebo | Metformin | 16 weeks | Deaths, volume depletion |
| **Sinclair 2016**[**^32^**](#_ENREF_32) | T2DM | 4058 | 2364 | 58.2 | 8.1% | Canagliﬂozin 100mg, 300mg | | Placebo | - | 52 weeks | Deaths, volume depletion |
| **Søfteland 2017**[**^33^**](#_ENREF_33) | T2DM | 327 | 191 | 55.2 | 7.97% | Empagliflozin 10 mg, 25 mg | | Placebo | Metformin+ Linagliptin | 24 weeks | Deaths |
| **Stenlo¨f 2014**[**^34^**](#_ENREF_34) | T2DM | 584 | 258 | 55.4 | 8.0% | Canagliﬂozin 100mg, 300mg | | Placebo | - | 52 weeks | Deaths, volume depletion |
| **Strojek 2014**[**^35^**](#_ENREF_35) | T2DM | 592 | 285 | 59.8 | 8.11% | Dapagliflozin 2.5mg, 5mg, 10mg | | Placebo | Glimepiride | 48 weeks | Deaths, cardiovascular events, volume depletion |
| **Tikkanen 2015**[**^36^**](#_ENREF_36) | T2DM and hypertension | 823 | 495 | 60.2 | 7.90% | Empagliflozin 10 mg, 25 mg | | Placebo | - | 12 weeks | Deaths  Volume depletion |
| **Tinahones 2016**[**^37^**](#_ENREF_37) | T2DM | 467 | 254 | 56.6 | 7.96% | Empagliflozin 10 mg, 25 mg | | Placebo | Metformin+ Linagliptin | 24 weeks | Deaths |
| **Weber 2016**[**^38^**](#_ENREF_38) | T2DM and hypertension | 449 | 247 | 56.6 | 8.05% | Dapagliflozin 10mg | | Placebo | - | 12 weeks | Volume depletion |
| **Wilding 2013**[**^39^**](#_ENREF_39) | T2DM | 469 | 239 | 56.8 | 8.10% | Canagliﬂozin 100mg, 300mg | | Placebo | - | 52 weeks | Deaths  Volume depletion |
| **Wiviott 2019**[**^40^**](#_ENREF_40) | T2DM | 17160 | 10738 | 63.7 | 8.30% | Dapagliflozin 10mg | | Placebo | - | 201 weeks | Deaths, cardiovascular events, volume depletion |
| **Yale 2014**[**^41^**](#_ENREF_41) | T2DM and CKD | 269 | 163 | 68.5 | 8.0% | Canagliﬂozin 100mg, 300mg | | Placebo | - | 52 weeks | Deaths, volume depletion |
| **Yale 2017**[**^42^**](#_ENREF_42) | T2DM | 146 | 79 | 65.1 | 8.2% | Canagliﬂozin 100mg, 300mg | | Placebo | - | 52 weeks | Deaths, volume depletion |
| **Yang 2017**[**^43^**](#_ENREF_43) | T2DM | 1453 | 801 | 54.7 | 8.12% | Dapagliflozin 5mg, 10mg | | Placebo | - | 24 weeks | Deaths, volume depletion |
| **Yang 2018**[**^44^**](#_ENREF_44) | T2DM | 272 | 110 | 57.5 | 8.56% | Dapagliflozin 10mg | | Placebo | Insulin | 24 weeks | Deaths, volume depletion |
| **Zinman 2015**[**^45^**](#_ENREF_45) | T2DM | 7020 | 5016 | 63.1 | 8.7% | Empagliflozin 10mg, 25mg | | Placebo | - | 136 weeks | Deaths, cardiovascular events, volume depletion |
| **Mordi 2020**[**^46^**](#_ENREF_46) | T2DM, HF | 23 | 17 | 69.8 | 7.9% | Empagliflozin 25mg | | Placebo | furosemide | 8 weeks | Cardiovascular events  Volume depletion |
| **Eickhoff 2020**[**^47^**](#_ENREF_47) | T2DM | 36 | 89 | 64.0 | 7.5% | Dapagliflozin 10 mg | | Placebo | - | 24 weeks | Deaths, cardiovascular events, volume depletion |

Abbreviations: RCTs, randomized controlled trial; N, mumble; HbA1C, hemoglobin A1c; SGLT2, Sodium glucose co-transporter 2; T2DM, type 2 diabetes mellitus; CKD, chronic kidney disease; CVD, Cardiovascular disease.

**Reference:**

1. Bailey CJ, Gross JL, Hennicken D, Iqbal N, Mansfield TA, List JF. Dapagliflozin add-on to metformin in type 2 diabetes inadequately controlled with metformin: a randomized, double-blind, placebo-controlled 102-week trial. *BMC medicine.* Feb 20 2013;11:43.

2. Bailey CJ, Morales Villegas EC, Woo V, Tang W, Ptaszynska A, List JF. Efficacy and safety of dapagliflozin monotherapy in people with Type 2 diabetes: a randomized double-blind placebo-controlled 102-week trial. *Diabetic medicine : a journal of the British Diabetic Association.* Apr 2015;32(4):531-541.

3. Barnett AH, Mithal A, Manassie J, et al. Efficacy and safety of empagliflozin added to existing antidiabetes treatment in patients with type 2 diabetes and chronic kidney disease: a randomised, double-blind, placebo-controlled trial. *The lancet. Diabetes & endocrinology.* May 2014;2(5):369-384.

4. Bolinder J, Ljunggren O, Kullberg J, et al. Effects of dapagliflozin on body weight, total fat mass, and regional adipose tissue distribution in patients with type 2 diabetes mellitus with inadequate glycemic control on metformin. *The Journal of clinical endocrinology and metabolism.* Mar 2012;97(3):1020-1031.

5. Bode B, Stenlof K, Harris S, et al. Long-term efficacy and safety of canagliflozin over 104 weeks in patients aged 55-80 years with type 2 diabetes. *Diabetes, obesity & metabolism.* Mar 2015;17(3):294-303.

6. Davies MJ, Merton K, Vijapurkar U, Yee J, Qiu R. Efficacy and safety of canagliflozin in patients with type 2 diabetes based on history of cardiovascular disease or cardiovascular risk factors: a post hoc analysis of pooled data. *Cardiovascular diabetology.* Mar 21 2017;16(1):40.

7. DeFronzo RA, Lewin A, Patel S, et al. Combination of empagliflozin and linagliptin as second-line therapy in subjects with type 2 diabetes inadequately controlled on metformin. *Diabetes care.* Mar 2015;38(3):384-393.

8. Fioretto P, Del Prato S, Buse JB, et al. Efficacy and safety of dapagliflozin in patients with type 2 diabetes and moderate renal impairment (chronic kidney disease stage 3A): The DERIVE Study. *Diabetes, obesity & metabolism.* Nov 2018;20(11):2532-2540.

9. Ferrannini E, Seman L, Seewaldt-Becker E, Hantel S, Pinnetti S, Woerle HJ. A Phase IIb, randomized, placebo-controlled study of the SGLT2 inhibitor empagliflozin in patients with type 2 diabetes. *Diabetes, obesity & metabolism.* Aug 2013;15(8):721-728.

10. Frias JP, Guja C, Hardy E, et al. Exenatide once weekly plus dapagliflozin once daily versus exenatide or dapagliflozin alone in patients with type 2 diabetes inadequately controlled with metformin monotherapy (DURATION-8): a 28 week, multicentre, double-blind, phase 3, randomised controlled trial. *The lancet. Diabetes & endocrinology.* Dec 2016;4(12):1004-1016.

11. Fulcher G, Matthews DR, Perkovic V, et al. Efficacy and safety of canagliflozin when used in conjunction with incretin-mimetic therapy in patients with type 2 diabetes. *Diabetes, obesity & metabolism.* Jan 2016;18(1):82-91.

12. Haering HU, Merker L, Christiansen AV, et al. Empagliflozin as add-on to metformin plus sulphonylurea in patients with type 2 diabetes. *Diabetes research and clinical practice.* Oct 2015;110(1):82-90.

13. Henry RR, Strange P, Zhou R, et al. Effects of Dapagliflozin on 24-Hour Glycemic Control in Patients with Type 2 Diabetes: A Randomized Controlled Trial. *Diabetes technology & therapeutics.* Nov 2018;20(11):715-724.

14. Inagaki N, Harashima S, Maruyama N, Kawaguchi Y, Goda M, Iijima H. Efficacy and safety of canagliflozin in combination with insulin: a double-blind, randomized, placebo-controlled study in Japanese patients with type 2 diabetes mellitus. *Cardiovascular diabetology.* Jun 18 2016;15:89.

15. Jabbour SA, Frias JP, Hardy E, et al. Safety and Efficacy of Exenatide Once Weekly Plus Dapagliflozin Once Daily Versus Exenatide or Dapagliflozin Alone in Patients With Type 2 Diabetes Inadequately Controlled With Metformin Monotherapy: 52-Week Results of the DURATION-8 Randomized Controlled Trial. *Diabetes care.* Oct 2018;41(10):2136-2146.

16. Kadowaki T, Haneda M, Inagaki N, et al. Empagliflozin monotherapy in Japanese patients with type 2 diabetes mellitus: a randomized, 12-week, double-blind, placebo-controlled, phase II trial. *Advances in therapy.* Jun 2014;31(6):621-638.

17. Kaku K, Kiyosue A, Inoue S, et al. Efficacy and safety of dapagliflozin monotherapy in Japanese patients with type 2 diabetes inadequately controlled by diet and exercise. *Diabetes, obesity & metabolism.* Nov 2014;16(11):1102-1110.

18. Kaku K, Lee J, Mattheus M, et al. Empagliflozin and Cardiovascular Outcomes in Asian Patients With Type 2 Diabetes and Established Cardiovascular Disease- Results From EMPA-REG OUTCOME((R)). *Circulation journal : official journal of the Japanese Circulation Society.* Jan 25 2017;81(2):227-234.

19. Kohan DE, Fioretto P, Tang W, List JF. Long-term study of patients with type 2 diabetes and moderate renal impairment shows that dapagliflozin reduces weight and blood pressure but does not improve glycemic control. *Kidney international.* Apr 2014;85(4):962-971.

20. Kovacs CS, Seshiah V, Merker L, et al. Empagliflozin as Add-on Therapy to Pioglitazone With or Without Metformin in Patients With Type 2 Diabetes Mellitus. *Clinical therapeutics.* Aug 2015;37(8):1773-1788 e1771.

21. Lavalle-Gonzalez FJ, Eliaschewitz FG, Cerdas S, Chacon Mdel P, Tong C, Alba M. Efficacy and safety of canagliflozin in patients with type 2 diabetes mellitus from Latin America. *Current medical research and opinion.* 2016;32(3):427-439.

22. Leiter LA, Cefalu WT, de Bruin TW, Gause-Nilsson I, Sugg J, Parikh SJ. Dapagliflozin added to usual care in individuals with type 2 diabetes mellitus with preexisting cardiovascular disease: a 24-week, multicenter, randomized, double-blind, placebo-controlled study with a 28-week extension. *Journal of the American Geriatrics Society.* Jul 2014;62(7):1252-1262.

23. Mahaffey KW, Jardine MJ, Bompoint S, et al. Canagliflozin and Cardiovascular and Renal Outcomes in Type 2 Diabetes Mellitus and Chronic Kidney Disease in Primary and Secondary Cardiovascular Prevention Groups. *Circulation.* Aug 27 2019;140(9):739-750.

24. Merton K, Davies MJ, Vijapurkar U, Inman D, Meininger G. Achieving the composite endpoint of HbA1c, body weight, and systolic blood pressure reduction with canagliflozin in patients with type 2 diabetes. *Current medical research and opinion.* Feb 2018;34(2):313-318.

25. Perkovic V, Jardine MJ, Neal B, et al. Canagliflozin and Renal Outcomes in Type 2 Diabetes and Nephropathy. *The New England journal of medicine.* Jun 13 2019;380(24):2295-2306.

26. Roden M, Merker L, Christiansen AV, et al. Safety, tolerability and effects on cardiometabolic risk factors of empagliflozin monotherapy in drug-naive patients with type 2 diabetes: a double-blind extension of a Phase III randomized controlled trial. *Cardiovascular diabetology.* Dec 23 2015;14:154.

27. Romera I, Gomis R, Crowe S, et al. Empagliflozin in combination with oral agents in young and overweight/obese Type 2 diabetes mellitus patients: A pooled analysis of three randomized trials. *Journal of diabetes and its complications.* Nov - Dec 2016;30(8):1571-1576.

28. Rosenstock J, Chuck L, Gonzalez-Ortiz M, et al. Initial Combination Therapy With Canagliflozin Plus Metformin Versus Each Component as Monotherapy for Drug-Naive Type 2 Diabetes. *Diabetes care.* Mar 2016;39(3):353-362.

29. Rosenstock J, Seman LJ, Jelaska A, et al. Efficacy and safety of empagliflozin, a sodium glucose cotransporter 2 (SGLT2) inhibitor, as add-on to metformin in type 2 diabetes with mild hyperglycaemia. *Diabetes, obesity & metabolism.* Dec 2013;15(12):1154-1160.

30. Rosenstock J, Jelaska A, Zeller C, et al. Impact of empagliflozin added on to basal insulin in type 2 diabetes inadequately controlled on basal insulin: a 78-week randomized, double-blind, placebo-controlled trial. *Diabetes, obesity & metabolism.* Oct 2015;17(10):936-948.

31. Schumm-Draeger PM, Burgess L, Koranyi L, Hruba V, Hamer-Maansson JE, de Bruin TW. Twice-daily dapagliflozin co-administered with metformin in type 2 diabetes: a 16-week randomized, placebo-controlled clinical trial. *Diabetes, obesity & metabolism.* Jan 2015;17(1):42-51.

32. Sinclair AJ, Bode B, Harris S, et al. Efficacy and Safety of Canagliflozin in Individuals Aged 75 and Older with Type 2 Diabetes Mellitus: A Pooled Analysis. *Journal of the American Geriatrics Society.* Mar 2016;64(3):543-552.

33. Softeland E, Meier JJ, Vangen B, Toorawa R, Maldonado-Lutomirsky M, Broedl UC. Empagliflozin as Add-on Therapy in Patients With Type 2 Diabetes Inadequately Controlled With Linagliptin and Metformin: A 24-Week Randomized, Double-Blind, Parallel-Group Trial. *Diabetes care.* Feb 2017;40(2):201-209.

34. Stenlof K, Cefalu WT, Kim KA, et al. Long-term efficacy and safety of canagliflozin monotherapy in patients with type 2 diabetes inadequately controlled with diet and exercise: findings from the 52-week CANTATA-M study. *Current medical research and opinion.* Feb 2014;30(2):163-175.

35. Strojek K, Yoon KH, Hruba V, Sugg J, Langkilde AM, Parikh S. Dapagliflozin added to glimepiride in patients with type 2 diabetes mellitus sustains glycemic control and weight loss over 48 weeks: a randomized, double-blind, parallel-group, placebo-controlled trial. *Diabetes therapy : research, treatment and education of diabetes and related disorders.* Jun 2014;5(1):267-283.

36. Tikkanen I, Narko K, Zeller C, et al. Empagliflozin reduces blood pressure in patients with type 2 diabetes and hypertension. *Diabetes care.* Mar 2015;38(3):420-428.

37. Tinahones FJ, Gallwitz B, Nordaby M, et al. Linagliptin as add-on to empagliflozin and metformin in patients with type 2 diabetes: Two 24-week randomized, double-blind, double-dummy, parallel-group trials. *Diabetes, obesity & metabolism.* Feb 2017;19(2):266-274.

38. Weber MA, Mansfield TA, Cain VA, Iqbal N, Parikh S, Ptaszynska A. Blood pressure and glycaemic effects of dapagliflozin versus placebo in patients with type 2 diabetes on combination antihypertensive therapy: a randomised, double-blind, placebo-controlled, phase 3 study. *The lancet. Diabetes & endocrinology.* Mar 2016;4(3):211-220.

39. Wilding JP, Charpentier G, Hollander P, et al. Efficacy and safety of canagliflozin in patients with type 2 diabetes mellitus inadequately controlled with metformin and sulphonylurea: a randomised trial. *International journal of clinical practice.* Dec 2013;67(12):1267-1282.

40. Wiviott SD, Raz I, Bonaca MP, et al. Dapagliflozin and Cardiovascular Outcomes in Type 2 Diabetes. *The New England journal of medicine.* Jan 24 2019;380(4):347-357.

41. Yale JF, Bakris G, Cariou B, et al. Efficacy and safety of canagliflozin over 52 weeks in patients with type 2 diabetes mellitus and chronic kidney disease. *Diabetes, obesity & metabolism.* Oct 2014;16(10):1016-1027.

42. Yale JF, Xie J, Sherman SE, Garceau C. Canagliflozin in Conjunction With Sulfonylurea Maintains Glycemic Control and Weight Loss Over 52 Weeks: A Randomized, Controlled Trial in Patients With Type 2 Diabetes Mellitus. *Clinical therapeutics.* Nov 2017;39(11):2230-2242 e2232.

43. Yang W, Ji L, Zhou Z, Cain VA, Johnsson KM, Sjostrom CD. Efficacy and safety of dapagliflozin in Asian patients: A pooled analysis. *Journal of diabetes.* Aug 2017;9(8):787-799.

44. Yang W, Ma J, Li Y, et al. Dapagliflozin as add-on therapy in Asian patients with type 2 diabetes inadequately controlled on insulin with or without oral antihyperglycemic drugs: A randomized controlled trial. *Journal of diabetes.* Jul 2018;10(7):589-599.

45. Zinman B, Wanner C, Lachin JM, et al. Empagliflozin, Cardiovascular Outcomes, and Mortality in Type 2 Diabetes. *The New England journal of medicine.* Nov 26 2015;373(22):2117-2128.

46. Mordi NA, Mordi IR, Singh JS, McCrimmon RJ, Struthers AD, Lang CC. Renal and Cardiovascular Effects of SGLT2 Inhibition in Combination with Loop Diuretics in Patients with Type 2 Diabetes and Chronic Heart Failure: The RECEDE-CHF Trial. *Circulation.* Aug 29 2020.

47. Eickhoff MK, Olsen FJ, Frimodt-Moller M, et al. Effect of dapagliflozin on cardiac function in people with type 2 diabetes and albuminuria - A double blind randomized placebo-controlled crossover trial. *Journal of diabetes and its complications.* Jul 2020;34(7):107590.

Table S3. Risk of bias of included studies.

| **Study, Year** | **Selection Bias** | | **Information Bias** | | | **Analysis Bias** | | **Sum bias** |
| --- | --- | --- | --- | --- | --- | --- | --- | --- |
|  | Adequate Sequence Generation | Allocation Concealment | Masking (deaths) | Masking (cardiovascular event) | Masking (volume depletion) | Intention to Treat Analysis | Loss of Follow-up |  |
| Bailey 2013[^1^](#_ENREF_1) | Low | Low | Unclear | Unclear | Low | Low | Low | Low |
| Bailey 2015[^2^](#_ENREF_2) | Low | Low | Low | Low | Low | Low | Low | Low |
| Barnett 2014[^3^](#_ENREF_3) | Low | Low | Low | Low | Low | Low | Unclear | Low |
| Bolinder 2012[^4^](#_ENREF_4) | Low | Low | Low | Low | Low | Low | Low | Low |
| Bode 2015[^5^](#_ENREF_5) | Low | Low | Low | Low | Low | Low | Low | Low |
| Davies 2017[^6^](#_ENREF_6) | Low | Low | Low | Low | Low | Low | Low | Low |
| DeFronzo 2015[^7^](#_ENREF_7) | Low | Low | Low | Low | Low | Low | Low | Low |
| Fioretto 2018[^8^](#_ENREF_8) | Low | Low | Low | Low | Low | Low | Low | Low |
| Ferrannini 2013[^9^](#_ENREF_9) | Low | Low | Low | Low | Low | Low | Low | Low |
| Frías 2016[^10^](#_ENREF_10) | Unclear | Unclear | Low | Low | Low | Unclear | Low | Unclear |
| Fulcher 2015[^11^](#_ENREF_11) | Unclear | Unclear | Low | Unclear | Low | Unclear | Low | Unclear |
| Haring 2015[^12^](#_ENREF_12) | Unclear | Unclear | Low | Low | Low | Unclear | Low | Unclear |
| Henry 2018[^13^](#_ENREF_13) | Low | Low | Low | Low | Low | Low | Low | Low |
| Inagaki 2016[^14^](#_ENREF_14) | Low | Low | Low | Low | Low | Low | Low | Low |
| Jabbour 2018[^15^](#_ENREF_15) | Low | Low | Low | Low | Low | Low | Low | Low |
| Kadowaki 2014[^16^](#_ENREF_16) | Unclear | Unclear | Low | Low | Low | Unclear | Low | Unclear |
| Kaku 2014[^17^](#_ENREF_17) | Unclear | Unclear | Low | Unclear | Low | Unclear | Low | Unclear |
| Kohan 2014[^19^](#_ENREF_19) | Low | Low | Low | Low | Low | Low | Low | Low |
| Kovacs 2015[^20^](#_ENREF_20) | Unclear | Unclear | Low | Low | Low | Unclear | Low | Unclear |
| Lavalle-González 2016[^21^](#_ENREF_21) | Unclear | Unclear | Low | Unclear | Low | Unclear | Low | Unclear |
| Leiter 2014[^22^](#_ENREF_22) | Low | Low | Low | Low | Low | Low | Low | Low |
| Mahaffey 2019[^23^](#_ENREF_23) | Unclear | Unclear | Low | Low | Low | Unclear | Low | Unclear |
| Merton 2018[^24^](#_ENREF_24) | Unclear | Unclear | Low | Unclear | Low | Unclear | Low | Unclear |
| Perkovic 2019[^25^](#_ENREF_25) | Low | Low | Low | Low | Low | Low | Low | Low |
| Roden 20158[^26^](#_ENREF_26) | Low | Low | Low | Low | Low | Low | Low | Low |
| Romera 2016[^27^](#_ENREF_27) | Low | Low | Low | Low | Low | Low | Low | Low |
| Rosenstock 2013[^29^](#_ENREF_29) | Low | Low | Low | Low | Low | Low | Low | Low |
| Rosenstock 2016[^28^](#_ENREF_28) | Unclear | Unclear | Low | Low | Low | Unclear | Low | Unclear |
| Rosenstock 2015[^30^](#_ENREF_30) | Unclear | Unclear | Low | Unclear | Low | Unclear | Low | Unclear |
| Schumm-Draeger 2016[^31^](#_ENREF_31) | Low | Low | Low | Low | Low | Low | Low | Low |
| Søfteland 2017[^33^](#_ENREF_33) | Unclear | Unclear | Low | Unclear | Low | Unclear | Low | Unclear |
| Stenlo¨f 2014[^34^](#_ENREF_34) | Low | Low | Low | Low | Low | Low | Low | Low |
| Strojek 2014[^35^](#_ENREF_35) | Unclear | Unclear | Low | Low | Low | Unclear | Low | Unclear |
| Tikkanen 2015[^36^](#_ENREF_36) | Unclear | Unclear | Low | Low | Low | Unclear | Low | Unclear |
| Tinahones 2016[^37^](#_ENREF_37) | Unclear | Unclear | Low | Unclear | Low | Unclear | Low | Unclear |
| Weber 2016[^38^](#_ENREF_38) | Unclear | Unclear | Low | Low | Low | Unclear | Low | Unclear |
| Wilding 2013[^39^](#_ENREF_39) | Unclear | Unclear | Low | Unclear | Low | Unclear | Low | Unclear |
| Wiviott 2019[^40^](#_ENREF_40) | Low | Low | Low | Low | Low | Low | Low | Low |
| Yale 2014[^41^](#_ENREF_41) | Unclear | Unclear | Low | Low | Low | Unclear | Low | Unclear |
| Yale 2017[^42^](#_ENREF_42) | Unclear | Unclear | Low | Unclear | Low | Unclear | Low | Unclear |
| Yang 2017[^43^](#_ENREF_43) | Low | Low | Low | Low | Low | Low | Low | Low |
| Yang 2018[^44^](#_ENREF_44) | Low | Low | Low | Low | Low | Low | Low | Low |
| Zinman 2015[^45^](#_ENREF_45) | Low | Low | Low | Low | Low | Low | Low | Low |
| Mordi 2020[^46^](#_ENREF_46) | Unclear | Unclear | Low | Low | Low | Unclear | Low | Unclear |
| Eickhoff 2020[^47^](#_ENREF_47) | Unclear | Unclear | Low | Unclear | Low | Unclear | Low | Unclear |

Figure S1. Inconsistency

Figure S1A. Inconsistency for all-cause death. (1, placebo; 2, dapagliflozin 2.5mg; 3, dapagliflozin 5mg; 4, dapagliflozin 10mg; 5, empagliflozin 10mg; 6, empagliflozin 25mg; 7, canagliflozin 100mg; 8, canagliflozin 300mg)

Figure S1B. Inconsistency for cardiovascular events. (1, placebo; 2, dapagliflozin 2.5mg; 3, dapagliflozin 5mg; 4, dapagliflozin 10mg; 5, empagliflozin 10mg; 6, empagliflozin 25mg; 7, canagliflozin 100mg; 8, canagliflozin 300mg)

Figure S1C. Inconsistency using the node-splitting approach for volume depletion. (1, placebo; 2, dapagliflozin 2.5mg; 3, dapagliflozin 5mg; 4, dapagliflozin 10mg; 5, empagliflozin 10mg; 6, empagliflozin 25mg; 7, canagliflozin 100mg; 8, canagliflozin 300mg)

**Figure S2**. Publication bias of funnel plot

**Figure S2**-A: funnel plot for all-cause death. (1, placebo; 2, dapagliflozin 2.5mg; 3, dapagliflozin 5mg; 4, dapagliflozin 10mg; 5, empagliflozin 10mg; 6, empagliflozin 25mg; 7, canagliflozin 100mg; 8, canagliflozin 300mg)

**Figure S2**-B: funnel plot for cardiovascular events. (1, placebo; 2, dapagliflozin 2.5mg; 3, dapagliflozin 5mg; 4, dapagliflozin 10mg; 5, empagliflozin 10mg; 6, empagliflozin 25mg; 7, canagliflozin 100mg; 8, canagliflozin 300mg)

**Figure S2**-C: funnel plot for volume depletion. (1, placebo; 2, dapagliflozin 2.5mg; 3, dapagliflozin 5mg; 4, dapagliflozin 10mg; 5, empagliflozin 10mg; 6, empagliflozin 25mg; 7, canagliflozin 100mg; 8, canagliflozin 300mg)
